# A Telephone-Based Tobacco Cessation Program in the State of Qatar: Protocol of a Feasibility Study

**DOI:** 10.3390/ijerph18094750

**Published:** 2021-04-29

**Authors:** Mohammed Al Thani, Vasiliki Leventakou, Angeliki Sofroniou, Safa M. Eltayeb, Eman Sadoun, Iman A. Hakim, Cynthia Thomson, Uma Nair

**Affiliations:** 1 Public Health Department, Ministry of Public Health, Doha P.O. Box 42, Qatar; malthani@moph.gov.qa (M.A.T.); asofroniou@moph.gov.qa (A.S.); seltayeb@moph.gov.qa (S.M.E.); 2 Health Research Governance Department, Ministry of Public Health, Doha P.O. Box 42, Qatar; esadoun@gmail.com; 3 Mel and Enid Zuckerman College of Public Health, University of Arizona, Tucson, AZ 85724, USA; ihakim@arizona.edu (I.A.H.); cthomson@arizona.edu (C.T.); umanair@arizona.edu (U.N.); 4 Family and Community Medicine, College of Medicine, University of Arizona, Tucson, AZ 85721, USA

**Keywords:** smoking quitline, Middle East, feasibility study, cognitive behavioral therapy, public health intervention

## Abstract

In Qatar, tobacco is the leading preventable cause of death and disease. Telephone-based interventions for smoking are cost-effective and scalable interventions that are effective in promoting smoking behavior change. While many countries have implemented these services within their tobacco control programs, there is a distinct dearth of a telephone-based smoking cessation intervention that is adapted and tailored to meet the needs of people who smoke in Qatar. This study presents the protocol of a primary health care center integrated smoking quitline program in Qatar. Participants will be recruited from seven smoking clinics (recruitment sites). Trained clinic staff will provide brief advice on quitting followed by a referral to the quitline. Eligible participants (male smokers over 18 years of age) will complete baseline questionnaires and receive five weekly proactive counseling calls, an end-of-treatment assessment (approx. 1 week after Session 5), and 1- and 3-month follow-up assessments. The main aim of this study is to assess the feasibility and acceptability, which include the recruitment and retention rate, compliance to pharmacotherapy, and participant satisfaction. This is the first study to integrate an evidence-based smoking cessation intervention delivered via telephone within the healthcare system in Qatar. If effective, results can inform the development of a large-scale telephone-based program that widely reaches users of tobacco in Qatar as well as in the Middle East.

## 1. Introduction

Tobacco is the leading preventable cause of death and disease contributing to approximately 8 million deaths per year globally of which more than 7 million are directly related to tobacco use [1]. Cigarette smoking continues to be the most common form of tobacco use [2]. In Qatar, approximately 28% of adult men and 1.5% of women report smoking tobacco based upon 2018 estimates [3], with smoking-related diseases (e.g., ischemic heart disease, lung cancer) being highly prevalent in the country [4,5]. To control tobacco use, Qatar, along with other countries, has adopted the WHO Framework Convention on Tobacco Control [6]. Within this framework, countries have committed to adopting effective measures for tobacco control (e.g., price and tax increases, smoke-free policies, bans on advertising) and providing tobacco intervention services that have a broad reach such as the telephone-based smoking cessation quitlines [7,8]. While Qatar has successfully implemented the above measures, there has been little focus on telephone-based behavioral counseling services for smoking cessation, and prevalence rates continue to remain high [3].

Quitlines are widely used as a population-oriented strategy for tobacco cessation with several advantages [8,9]. In the early 1980s, state-wide quitlines were established in the United States, followed by call-in centers in Australia and UK. Today, quitlines have become prevalent across North America, Europe, Australia, New Zealand, and Canada [7]. Tobacco users may receive counseling for assistance with quitting smoking with easy access (e.g., avoiding transportation and cost) and reduced logistical barriers. Quitlines can also be easily accessed by underserved populations who are unable to follow face-to-face cessation treatments (e.g., people of low socioeconomic status, elderly) [10,11], thereby increasing reach. Phone counseling may additionally facilitate discussions due to the protection of anonymity and increase likelihood of participant’s retention in the follow-up sessions, and when applying a structured protocol, they can provide individualized treatment [10]. Recent reports indicate that quitline services in the United States achieved a 30.3% cessation rate in 2018 [12]. Clients enrolled in quitline services receive a set number of proactive (counselor-initiated) and/or reactive calls (client-initiated) in order to develop behavioral and cognitive coping skills to address urge management, set a quit date, increase self-efficacy or confidence to carry out a personalized cessation plan, and avoid relapse [13,14]. Available research provides evidence that adding quitline counseling to medication further increases the effectiveness of quitlines as compared to medication alone [15,16]. Despite their successful implementation and uptake as standard practice in Western countries, little research has examined the effectiveness of telephone-based quitline services in Qatar. A randomized control trial by El Hajj et al. in 2015 showed the efficacy of a pharmacist-delivered in-person smoking cessation intervention that focused on education and coping skills [17]. The study included three sessions out of which only the first provided behavioral counseling. Given the wider reach and advantages of telephone-based counseling compared to in-person counseling, as well as the efficacy of these services in other countries, there has been a distinct absence of studies examining the feasibility of a telephone-based smoking intervention in Qatar. Moreover, such a service allows for the employment of dedicated smoking cessation counselors, trained in evidence-based behavioral skills who focus on building capacity for smokers to quit over time.

The proposed study describes the development of the infrastructure for a smoking cessation quitline in Qatar, a Middle East country with unique population characteristics related to cultural practices, climate, and socioeconomic status and with free to low-cost medication coverage for all residents. The goal of the study is to (a) implement a referral system within the primary healthcare smoking clinics to refer potential smokers who want to quit to a telephone-based behavioral counseling program and (b) conduct a feasibility and preliminary efficacy study on a telephone-based smoking cessation program among residents reporting tobacco use in Qatar. In this paper, we describe the protocol for developing the telephone-based program, counselor training materials, healthcare provider referral setup, and recruitment process. Logistical aspects of the program will lead to evidence-informed protocol modifications for optimizing the delivery of the intervention when expanded at the national level. Further, the protocol may be used to guide the development of tobacco cessation programs for other Middle Eastern countries—an area with similar tobacco use behaviors and significantly high rates of tobacco-related morbidity and mortality.

## 2. Materials and Methods

### 2.1. Study Design

This study is being conducted by the Ministry of Public Health in Qatar in collaboration with the researchers at the University of Arizona Mel & Enid Zuckerman College of Public Health, Global Health Institute, and College of Medicine. This study will adopt an approach to counseling similar to that of Arizona’s state quitline (ASHLine) services. Taking into consideration that the ASHline quit counselors in 2016 managed a caseload of about 11 clients per week, the target sample size of 200 participants for this study is feasible for an available timespan of 18–20 months of recruitment and the availability of trained counselors as research staff. All eligible patients who visit the smoking clinics (recruitment sites) will be asked for their consent to participate in the study and will be enrolled upon acceptance. The study protocol was reviewed and approved by the Institutional Review Board (IRB) of the primary healthcare centers (PHCCs) in Qatar.

### 2.2. Development of Healthcare System Referrals

In Qatar, all residents are registered at a local primary healthcare center (PHCC) to receive fully integrated healthcare. There are 27 PHCCs in Doha and at least 7 have integrated smoking cessation clinics. Smokers who are interested in acquiring smoking cessation assistance are referred to the smoking clinics, where assistance is limited to the provision of pharmacotherapy that includes Champix (varenicline) and/or provision of nicotine replacement therapy (gum, patch, or lozenge). The medication is provided by the pharmacies established within each healthcare center, and the cost is fully covered for nationals and 75% covered for residents by the national healthcare system. This paper describes the integration of a behavioral counseling program within the existing infrastructure of the smoking clinics.

**Figure 1:**
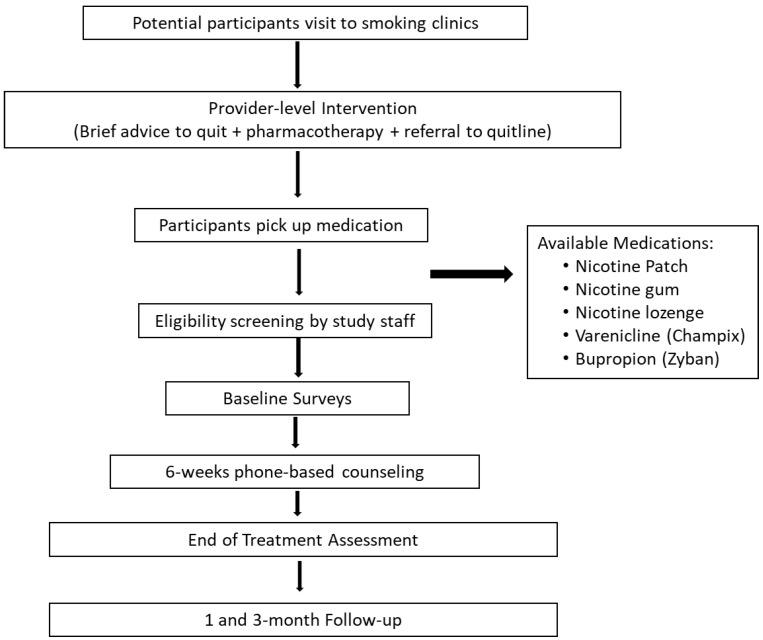
Participant recruitment and study flow.

Participants will be recruited from seven smoking clinics (recruitment sites). Prior to referring participants, clinic staff will receive training on (a) benefits of integrating behavioral counseling with cessation pharmacotherapy, (b) structure of the phone-based behavioral intervention, and (c) process for referring potential participants to the study. To facilitate the referral process, research staff will be present at the clinics to screen interested participants, thereby allowing for a seamless integration of provider advice to quit with enrollment into the phone-counseling intervention. Eligibility criteria include the following: be male current smokers (smoke at least one cigarette per day), be 18 years or above, speak and write English or Arabic with the ability to understand and comply with study procedures, have a valid phone number, and be willing to receive telephone coaching. Respecting social/cultural norms of the country, the study focused only on male smokers since it is culturally not widely accepted for women to smoke. Exclusion criteria include active psychosis, non-nicotine drug dependence, exclusive use of electronic cigarettes or electronic nicotine delivery system (ENDS), use of smokeless tobacco, and/or hookah or shisha use. All participants will be screened by the doctor for medical conditions that may be counter-indications for nicotine replacement therapy usage (e.g., history of heart attacks, angina, and chest pain) and will be prescribed medication accordingly (Figure 1). Eligible participants will provide written informed consent, complete baseline assessments, and receive an orientation to the behavioral counseling intervention prior to initiating the program. This will include a brief overview of the program, a timeline and schedule of calls, and staff will schedule the first phone session (approximately 24–48 h after baseline assessment) with an assigned quit counselor.

### 2.3. Counseling Intervention

Following standard quitline protocols, the counseling program consists of five weekly proactive counseling calls (as shown in Table 1). The counselors will guide participants to preparation for cessation through a behavioral shaping approach that reinforces motivation and confidence in achieving long-term smoking cessation outcomes. The first three sessions focus on increasing motivation to quit, developing cognitive and behavioral coping strategies to manage urges to smoke, understanding the trigger–urge connection, increasing adherence to the pharmacotherapy, and tips to prepare to quit smoking. Using a collaborative approach, counselors will encourage participants to quit in week 3 of the program, with sessions 4 and 5 focusing on relapse prevention and continued adherence to cessation medication. At the end of the counseling program (at session 5), counselors will schedule an end-of-treatment assessment (approx. 1 week after Session 5). Follow-up sessions will occur at 1 and 3 months after the end-of-treatment assessment.

### 2.4. Counselor Training and Fidelity

Study counselors will be trained in evidence-based approaches to smoking cessation that will include cognitive behavioral treatment and motivational interviewing techniques. Motivational interviewing aims to help people who smoke explore and resolve their ambivalence about smoking behavior change. This focuses on motivational processes that can enable behavior change of the individual and is highly related to cessation success [18]. Additionally, a cognitive behavioral treatment approach that involves the use of techniques such as self-regulation and identification of smoking triggers and works with the smokers to develop alternate healthy coping strategies (use of stimulus and urge control strategies) is applied. Specific training topics will include support to prepare participants for a quit day, increasing motivation to quit, social support, and information, education on smoking cessation medication (e.g., usage, side-effects monitoring, and strategies to increase medication adherence) and the physiology of tobacco addiction and dependence. Training will occur using didactic sessions, review of current literature, and role-playing sessions. Upon completion of training, counselors will be independently evaluated by two co-investigators who will listen to the session recordings (on a monthly basis) to assess fidelity and competency using a standardized fidelity checklist (e.g., active listening, motivational interviewing, tobacco use, empathy, use of cognitive behavioral strategies). Counseling materials will be translated into Arabic by a bilingual translator (native Arabic speaker) and examined by the investigator team fluent in Arabic for cultural competency.

### 2.5. Data Collection and Measures

Assessment data (baseline, weekly sessions, end of treatment, and 1- and 3-month follow-up sessions) will be entered and maintained electronically on REDCap (a HIPAA-compliant database system). Baseline assessments will include demographic characteristics (age, marital status, level of education, employment status, and annual income), smoking history (number of smoking years, history of previous quit attempts, past trials of smoking cessation, and intention of quitting in the next 6 months), and smoking practices at home (home smoking rules and smoking among family members; use of e-cigarette or vaping products). Smoker’s psychosocial characteristics (nicotine dependence, self-efficacy, urge coping, and depressive symptoms) will also be assessed at baseline.

The Fagerström Test of Nicotine Dependence will be used to measure smokers’ level of nicotine dependence [19,20]. Smoking self-efficacy will be assessed using a 12-item questionnaire to measure confidence in the participant’s ability to refrain from smoking when faced with internal (e.g., feeling nervous, happy, depressed) and external stimuli (e.g., being with other smokers) [21]. Smoking urges will be assessed using a 12-item scale adapted by O’Connell et al. [22], and people who smoke will be asked how often they used any of the reported coping strategies to prevent themselves from smoking a cigarette when they experienced a craving during the past week. The Center for Epidemiologic Studies Depression Scale [23] will be used to assess depressive symptoms that participants experienced over the past seven days. Participants will report the frequency of feelings and/or behaviors during the past week. The translation of the questionnaires (available in English and Arabic) will be conducted in advance using internationally recommended methodology [24], forward and backward translation; instruments will be pilot tested and adapted to the cultural norms and attitudes of the Qatar population, prior to use.

Weekly sessions (sessions 1–5) will include pharmacotherapy use, quit date and modifications, triggers, coping strategies, and challenges to behavior change. At the end of treatment and during 1- and 3-month follow-up sessions, data on the use of pharmacotherapy, quit status, and abstinence at 24 h, 7 days, and 30 days preceding the follow-up call will be recorded.

### 2.6. Study Outcomes

The primary objective of this study is to assess the feasibility and acceptability of a smoking cessation quitline, which include the following outcomes: (a) recruitment rate (i.e., number of participants enrolled per month), (b) retention rate (i.e., percent retained through the end of treatment and at 1- and 3-month follow-up assessments), (c) compliance to phone sessions, (d) compliance to pharmacotherapy, and (e) participant satisfaction at 1- and 3-month follow-up assessments. Secondary outcomes include self-reported abstinence prevalence at end of treatment and 1- and 3-month follow-up assessments. Finally, we will also examine referrals and treatment compliance for the seven clinics to identify differences in implementation across referral sites.

## 3. Conclusions

To our knowledge, this is the first study to describe the development of a telephone-based model, similar to a quitline, in the Middle East area that is embedded in the healthcare system. Current clinical guidelines recommend that healthcare providers assist patients to quit smoking by providing evidence-based counseling and monitoring follow-up [15]. However, physicians face barriers to providing individualized support and assistance to quitting due to lack of time, inadequate resources, insufficient smoking cessation skills, or sometimes lack of training to deliver the intervention [25,26]. Research shows that linking the physician with the quitline service increases the likelihood of smoking cessation [15,27,28]. Tobacco quitlines provide an excellent opportunity to support the healthcare system, remove the burden from healthcare providers, and improve the smoking cessation outcomes and related disease risks, in a cost-efficient manner. Lessons learned, feasibility, and acceptability of our intervention model, which integrates healthcare-system and population-level approaches, has immense potential for dissemination and implementation of evidence-based smoking cessation services across Qatar and, in the long run, in areas in the Middle East, where there is a dearth of evidence-based smoking cessation services. Given the variations in the available quitline services, it is important for such interventions to be empirically validated and standardized for the specific population characteristics before being implemented at a national level. These procedures may dramatically increase the effectiveness of the quitline and provide a strong degree of quality assurance via standardized training, structured protocols, and regular call monitoring. The current research will allow us to gain evidence for the counseling benefit throughout the intervention period.

There are limitations in this approach that merit consideration. Firstly, the study will not include bioverification of smoking abstinence at end of treatment and follow-up, due to additional costs and logistics of clinic-based integration of these measurements. The goal of this pilot study is to assess the acceptability and feasibility of a healthcare-system-integrated telephone-based smoking cessation program and, if promising, plans to expand the program by testing efficacy using bioverified quit outcomes in a research setting. Secondly, our counseling currently does not include cessation of exclusive use smokeless tobacco or shisha products. If successful, our model can be adapted to cover other forms of nicotine use.

Major efforts to improve smoking prevalence cannot rely solely on cessation interventions and efforts to increase reach. Advances in pharmacotherapy research accompanied by clinical guidelines, smoke-free policies, and cigarette prices may conjointly increase the cessation rate at a population level [6]. The proposed study provides a unique and cost-effective opportunity to leverage and adapt existing evidence-based cessation counseling in a quitline setting, which will allow the successful scalability and dissemination to a national level. Building upon the experience gained from this study, we will be able to expand the reach of the quitline and operate a national quitline service incorporated in the current healthcare system, accessible to all residents of Qatar. However, to achieve an effective population impact of the quitline, it will be necessary to build robust mechanisms and relationships between the quitline and health system, utilize appropriate models of quitline services (proactive and reactive system), optimize service operations, and continuously seek strategies to promote the services. The establishment of an effective telephone-based quitline model may serve as an effective treatment approach for other chronic health conditions such as cardiovascular health promotion, physical activity, and diet interventions.

## Figures and Tables

**Table 1:** Outline of quitline counseling sessions.

Sessions	Topics Covered
Session 1	Program orientation Exploring smoking history and previous quit attempts Setting a quit day in program NRT/pharmacotherapy overview Introduction to coping skills
Session 2	Understanding nicotine addiction Identifying triggers and high-risk situations Adding coping skills to tool kit Preparing to quit NRT/pharmacotherapy usage and adherence Increasing confidence to quit
Session 3 (Quit Day)	Review of withdrawal symptoms NRT/pharmacotherapy usage and adherence Preparing for being smoke-free Review of coping skills Motivation to stay smoke-free
Session 4	Review of withdrawal symptoms NRT/pharmacotherapy usage and adherence Relapse prevention
Session 5	NRT/pharmacotherapy usage and adherence Relapse prevention Staying an ex-smoker

## Data Availability

Not applicable.

## Abbreviations

ENDS	Electronic nicotine delivery system
HIPAA	Health Insurance Portability and Accountability Act
IRB	Institutional Review Board
PHCC	Primary healthcare centers
US	United States
UK	United Kingdom
